# Advances in the Design of pH-Sensitive Cubosome Liquid Crystalline Nanocarriers for Drug Delivery Applications

**DOI:** 10.3390/nano10050963

**Published:** 2020-05-18

**Authors:** Omar Mertins, Patrick D. Mathews, Angelina Angelova

**Affiliations:** 1 Institut Galien Paris-Saclay UMR8612, Université Paris-Saclay, CNRS, F-92296 Châtenay-Malabry, France; omar.mertins@universite-paris-saclay.fr; 2 Laboratory of Nano Bio Materials (LNBM), Department of Biophysics, Paulista Medical School, Federal University of Sao Paulo (UNIFESP), Sao Paulo 04023-062, Brazil; patrickmathews83@gmail.com; 3 Muséum National d’Histoire Naturelle, Sorbonne Université, CP 26, 75231 Paris, France

**Keywords:** pH-sensitive mesophase lipid structures, cubosomes with pH-responsive polymer shells, self-assembled lyotropic liquid crystalline nanoparticles, nonlamellar lipids, phase transitions, drug delivery vehicles, oral bioavailability

## Abstract

Nanostructure bicontinuous cubic phase self-assembled materials are receiving expanding applications as biocompatible delivery systems in various therapeutic fields. The functionalization of cubosome, spongosome, hexosome and liposome nanocarriers by pH-sensitive lipids and/or pH-sensitive polymer shells offers new opportunities for oral and topical drug delivery towards a new generation of cancer therapies. The electrochemical behavior of drug compounds may favor pH-triggered drug release as well. Here, we highlight recent investigations, which explore the phase behavior of mixed nonlamellar lipid/fatty acid or phospholipid systems for the design of pH-responsive and mucoadhesive drug delivery systems with sustained-release properties. X-ray diffraction and small-angle X-ray scattering (SAXS) techniques are widely used in the development of innovative delivery assemblies through detailed structural analyses of multiple amphiphilic compositions from the lipid/co-lipid/water phase diagrams. pH-responsive nanoscale materials and nanoparticles are required for challenging therapeutic applications such as oral delivery of therapeutic proteins and peptides as well as of poorly water-soluble substances. Perspective nanomedicine developments with smart cubosome nanocarriers may exploit compositions elaborated to overcome the intestinal obstacles, dual-drug loaded pH-sensitive liquid crystalline architectures aiming at enhanced therapeutic efficacy, as well as composite (lipid/polyelectrolyte) types of mucoadhesive controlled release colloidal cubosomal formulations for the improvement of the drugs’ bioavailability.

## 1. Introduction

Lipid nanoparticle-mediated drug delivery experiences rapid developments in the field of liquid crystalline colloidal carriers, e.g., cubosomes, spongosomes, hexosomes and vesicles [1,2,3,4,5,6,7]. In addition to the expansion of liposomes as advanced drug delivery systems [8,9,10], a plethora of research has been dedicated to diverse mesoporous liquid crystalline materials and nanostructures intended as drug delivery devices [11,12,13,14,15,16]. Lipid-based cubic mesophases are increasingly implemented in noninvasive drug delivery applications [17,18,19]. The liquid crystalline structure of cubosomes, consisting of well-defined networks of aqueous channels and lipid bilayer membranes, organized in periodic 3D topologies, presents advantages over other delivery systems [20]. Scientific and technological advances have been achieved in the fabrication of biocompatible systems for encapsulation of natural and synthetic lipophilic, hydrophilic and hydrophobic drug molecules, a variety of macromolecular drugs (peptides, proteins, DNA, siRNA, etc.) and imaging agents [1,2,3,4,5,6,7,11,12,13,14,15,16,17,18,19,21,22,23,24]. Complex cubic lattice networks of high surface areas provide enhanced protection of the incorporated payload from degradation as well as the prolonged and sustained release of the entrapped bioactive molecules [25,26]. 

**Figure 1:**
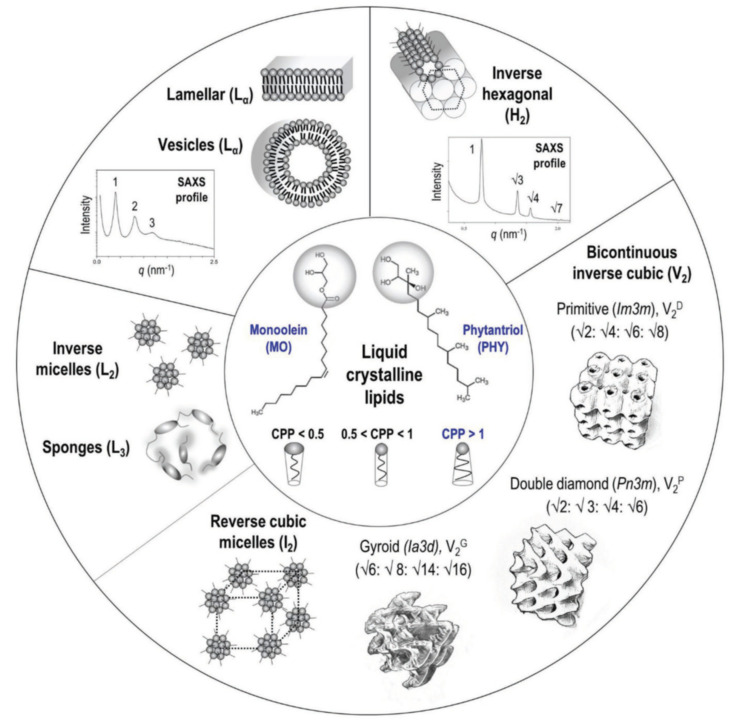
Structures of commonly encountered mesophases produced by the self-assembly of lyotropic lipids with varying critical packing parameters (CPP). The liquid crystalline phases are identified based on their characteristic diffraction spacing ratios determined by the small-angle X-ray scattering (SAXS) technique (reproduced from [7], with permission from Wiley-VCH Verlag GmbH & Co., 2020).

Current demands for improved performance and specificity of drug delivery carriers require the use of intelligent materials, which respond to various environmental stimuli [27]. In this context, the cubosome assemblies present further advantages because the transformations between the different liquid crystalline organizations, e.g., *Pn3m*, *Im3m* and *Ia3d*, besides the inverted hexagonal phases (Figure 1), can be tuned and controlled by changes in temperature, ionic strength or pH of the environment of the targeted application sites [28,29,30,31,32,33,34]. 

In the present work, we review recent advances in cubosome nanocarriers and bulk cubic mesophases with a particular emphasis on the pH effects on the structures and topologies of the designed drug delivery systems. This interest is motivated by the fact that pH represents an inherent physiological condition of all biological organisms and that pathological (inflamed or infected) areas can embed target sites for pH-sensitive nanomedicines. This has led to the emergence of pH-responsive drug delivery carriers and nanomaterials, some of which are reviewed here below.

**Figure 2:**
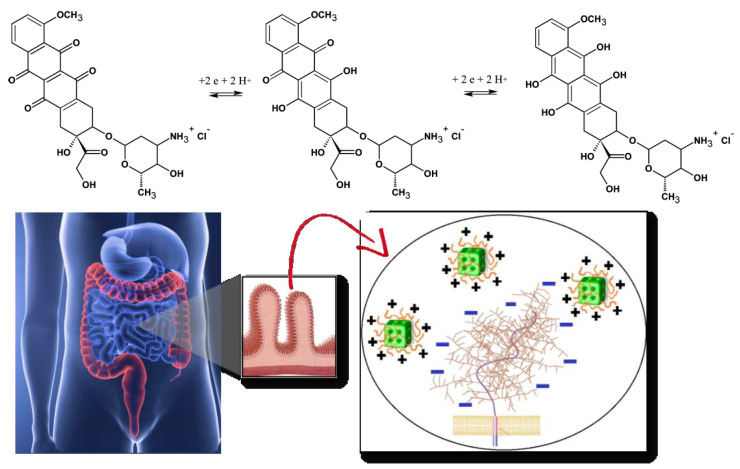
Chemical structures and redox process of the anticancer drug doxorubicin, from left to right: initial, oxidized and reduced forms of doxorubicin (top panel). Scheme of oral drug administration indicating the microvilli of the intestinal membrane, for which bioadhesive positively charged cubosome particles (modified by polyelectrolyte shells) interact with the negative charges of the mucin layer over the mucosal membrane (bottom panel). Either the biocompatible polyelectrolyte shells or the lipids, constituting the cubosome nanocarriers, are pH-sensitive.

Acidic compartments of the cells are the endosomes (pH 5–6) and the lysosomes (pH 5). Depending on the application site, pH changes of the biological medium may comprise a profitable condition to boost the target release of encapsulated drugs from the pH-responsive nanocarriers. In this context, lipid-based pH-sensitive cubosomes have been produced by the assembly of traditional cubic-phase-forming amphiphiles monoolein or phytantriol (Figure 1) with charged or ionizable lipids [35,36,37,38,39,40,41,42]. Incorporation of drugs like doxorubicin, for which the redox process (Figure 2) provokes pH-dependent structural changes, may also lead to pH-responsiveness of the host cubic phase carriers. Furthermore, the association of lipids with polyelectrolytes and charged surfactants was employed as a means to generate highly pH-sensitive cubosomes and other types of nanocarriers [43,44,45,46,47]. A summary of the published works, exploiting small-angle X-ray scattering (SAXS) analyses of liquid crystalline nanostructures and nanoparticles, is presented in Table 1 below. 

Oral delivery of peptides, recombinant proteins and other nanomedicines is of a primary therapeutic interest [48,49,50,51,52]. The oral drug administration represents several challenges. One of the most prominent is the considerable pH variation in the gastrointestinal tract (Figure 2, bottom panel). With this concern in mind, we discuss the perspectives of cubosomes development in oral drug delivery applications with special attention to composite nanocarriers, i.e., cubosomes with pH-responsive characteristics provided by polyelectrolyte shells [46,47,52]. The latter may ensure the structural stability of the carriers in adverse media, for instance under the strong acidic condition of the stomach. 

Increased oral drug bioavailability can be expected due to the mucoadhesive features of the cubic-phase forming lipids. Prolonged-release mucoadhesive formulations have been obtained thanks to the fusogenic properties of monoolein enabling permeation enhancer activity of the nanocarriers [1,17,18,19]. The mucoadhesive controlled release formulations interact with the mucosal components such as mucin (Figure 2, bottom panel). The enhanced adsorption on the intestinal epithelia ideally promotes a sustained drug release. 

Salentinig et al. have emphasized that the hydrolysis of monoolein in the zones of the gastrointestinal tract will produce oleic acid [37], the ionization state of which determines the structural properties of the carriers, through the lipid packing, and hence their fusion dynamics with the membranes [20].

pH-responsive lyotropic liquid crystalline phases and nanoparticles have received tremendous interest in view of applications in anticancer nanomedicine [3,4,11,18,28,29,31]. Although the pH of normal tissues and blood is around 7.4, the tumor environment exhibits acidic pH owing to the metabolic production of lactic acid under the conditions of fast cell growth and deficiency of oxygen and nutrients. The use of pH-sensitive cubosomes as drug delivery systems for cancer treatments presents an advantage of the pH difference between the tumor environment and the normal physiological condition [53,54]. In chemotherapy, pH-responsive drug delivery nanocarriers have been shown to accumulate in the tumor tissue via the enhanced permeability and retention (EPR) effect [53]. The release of anticancer drugs may be triggered in response to extracellular or intracellular chemical stimuli including pH-stimuli [53].

## 2. Charged Lipids as pH-Triggering Adjuvant 

Charged lipids have been used in several studies for electrostatic tuning of the cubosome internal organization and nanochannel sizes [55,56,57,58]. The incorporation of long-chain fatty acids has been broadly exploited for the preparation of pH-sensitive liquid crystalline assemblies [35,36,37,38,39,40,41,42]. The performed structural research, employing synchrotron radiation SAXS, has evidenced the potential of pH-induced hexagonal-to-cubic phase transitions for the development of anticancer drug delivery strategies [59,60,61,62,63,64,65,66,67,68,69]. 

**Figure 3:**
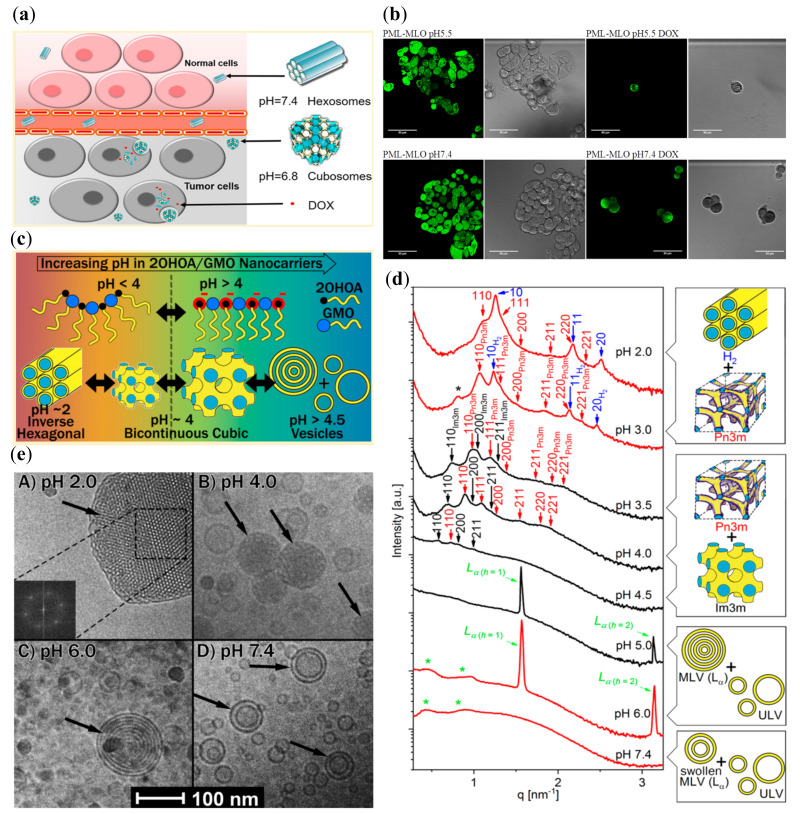
Examples of pH-induced phase transformations in self-assembled lipid-based nanostructures and related effects. (**a**) Opportunity for tumor targeting by pH-sensitive liquid crystalline nanocarriers dual-loaded by *Brucea javanica* oil (BJO) and doxorubicin (DOX; reproduced from [59], with permission from the American Chemical Society, 2020). (**b**) In vitro effect of a pyridinylmethyl linoleate-monolinolein (PML-MLO) liquid crystalline phase affecting HT29 human colon cancer cells in the absence or presence of DOX at two pH values (reproduced from [62], with permission from the Royal Society of Chemistry, 2020). (**c**) pH-dependent curvature of mixed monoolein (GMO)/2-hydroxyoleic acid (2OHOA) membranes causing phase transitions of the colloidal dispersions [63]; (**d**) SAXS patterns showing pH-induced phase transitions of the lipid structures studied in [63]; (**e**) Cryo-TEM images of colloidal nanocarrier dispersions with pH-dependent structures (reproduced from [63], with permission from the American Chemical Society, 2020).

Li et al. [59] used an increasing content of oleic acid to produce pH-sensitive cubosomes loaded with the anticancer drugs doxorubicin and *Brucea javanica* oil (BJO). The latter involves unsaturated fatty acids, the protonation/deprotonation equilibrium of which is pH-dependent and therefore advantageous for the fabrication of pH-responsive nanomedicines (Figure 3a). Using monoolein as the lipid matrix in conjunction with oleic acid, *Brucea javanica* oil served as a payload and Pluronic F127 as a stabilizer of the nanoformulations. The components were mixed by heating to 60 °C and a PBS buffer containing dissolved doxorubicin hydrochloride was subsequently added. Homogenization under high pressure was performed after intensive stirring in order to obtain dispersed dual-drug loaded nanoparticles (Table 1). The nanocarriers undergo a clear pH-induced structural switch of their inner liquid crystalline organizations [59]. Hexosomes with an inner hexagonal H_II_ phase structure were identified by SAXS at pH 7.4, whereas cubosomes with coexisting *Pn3m*/*Im3m* inner cubic structures formed at pH 6.8 and below. SAXS experiments established a transition of the inner crystalline organization to emulsified microemulsion at pH 5.3, which is related to changes in the interfacial hydration state of the lipid bilayers in the presence of oleic acid under acidic conditions. The higher concentrations of encapsulated *Brucea javanica* oil increased the fluidity and thus hampered the ordering of the lipid bilayers into a crystalline cubic lattice membrane (cubosome nanocarriers). In addition, the produced dual-drug loaded nanoparticles showed a shift to a decreased ordering of the internal structure at pH 7.4 upon loading of doxorubicin, while keeping the pH-responsive effect. The pH-induced phase change from an inverted hexagonal to a cubic network, and to a less ordered emulsified microemulsion phase, could be correlated with the sustained drug release properties of the nanoparticles. Moreover, in vitro studies of cytotoxicity and apoptosis with the human MCF-7 carcinoma cells demonstrated an enhanced antitumor activity, which was discussed in view of the pH-responsive properties of the cubosome particles (Figure 3a).

Nakano et al. [60] evaluated by SAXS the effect of oleic acid (OA) on the structure of blank monoolein-based cubosomes. The cubic phase *Im3m* diffraction pattern observed with the dispersed samples at pH 7 changed to that of an inverted hexagonal phase (H_II_) at pH 6, denoting the responsive effect of OA to the pH changes. The mean particle diameters of the dispersions were determined to be 140 nm at pH 7 and 204 nm at pH 6. However, when the pH decreased from seven to six, the mean diameter of the prepared cubosomes decreased to 125 nm. Meanwhile, the diffraction pattern of an emerging H_II_ structure was observed. The internal liquid crystalline structure varied in the order from bicontinuous cubic-to-inverted hexagonal-to-inverted cubic organization upon the increase of the oleic acid content. Therefore, both sizes of the particles and their internal structures were strongly dependent on pH. Actually, the decrease in pH increases the protonation of the oleic acid carboxylic headgroups, hence increasing the negative curvature of the membranes and decreasing their polarity. Evidently, the cubosome-to-hexosome transition is associated with the exclusion of water from the interior of the particles.

The cubic phase formed by monolinolein doped with linoleic acid (3 wt %) was characterized by the *Im3m* symmetry, for which a structural change was triggered from neutral pH to strong acidic conditions as described by Negrini and Mezzenga [61]. The incorporation of linoleic acid in the lipid’s bilayers at pH 7 was associated with an increased mean cross-sectional area of the lipid headgroups due to the deprotonated state of the carboxylates. The latter caused electrostatic self-repulsion in the polar regions of the bilayers, thus stabilizing the *Im3m* cubic symmetry. At pH 2, the electrostatic effect vanished with the protonation of the carboxylates, providing a decrease in the headgroup areas. Thus, the resulting increase of the critical packing parameter caused a phase transition from a cubic symmetry to an inverted hexagonal phase (H_II_). In this system, the diffusion of the encapsulated hydrophilic phloroglucinol drug occurred four times faster at pH 7 than at pH 2. It was further considered that when applying these concepts to cubosome particles of a few hundred nanometers in diameter, the phase transition may occur much faster than in the bulk liquid crystalline phase, leading to a sharper pH-induced release of encapsulated hydrophilic molecules.

A newly synthesized lipid pyridinylmethyl linoleate (PML) was incorporated in monolinolein (MLO) bilayers at a chosen percentage of 3.2 wt %, which induced pH-sensitive phase transition [62]. Negrini et al. exploited the fact that the amphiphilic PML molecule is a weak base, which is neutral at pH 7 and over, but positively charged under acidic pH conditions due to the protonation process [62]. The electrostatic self-repulsion and the resulting expansion of the effective headgroup area led to a change in the critical packing parameter of the amphiphile, respectively in changes in the lipid bilayer curvature. Thus, the lyotropic liquid crystalline matrix underwent a phase transition from a *Pn3m* cubic symmetry at pH 7.4 to an inverted hexagonal structure at pH 5.5. With doxorubicin as a payload, the study further demonstrated that the release of doxorubicin at pH 5.5 can be accelerated to a 10-fold faster release in an in vitro simulated tumor environment of HT29 human colon cancer cells. Only a minimal drug release occurred under normal physiological conditions. In addition, a three-fold increased efficiency was observed for cancer cells killing at pH 5.5 versus pH 7.4 (Figure 3b), which highlighted the investigated system as a prominent strategy for potential cancer treatments. 

The self-assembly of 2-hydroxyoleic acid (2OHOA) and monoolein was studied by Prajapati et al. towards the production of pH-responsive nanostructures [63]. At 1/1 mass ratio, the binary mixtures rendered cubosomes in excess aqueous buffer at pH values 4.5, 4.0 and 3.5. Whereas coexisting cubosomes and hexosomes were obtained at pH 3.0 and 2.0, mainly multilamellar and unilamellar vesicles formed at pH 5.0, 6.0 and 7.4 (Figure 3c,d). An inverted hexagonal phase (H_II_), along with a bicontinuous cubic structure of the *Pn3m* symmetry, was identified by SAXS under strong acidic conditions. A gradual evolvement of an *Im3m* cubic phase was established, while the H_II_ phase vanished in the intermediate pH region. The lattice spacing was found to increase with the pH increase. However, from pH 5.0 and over, the liquid crystalline structures transformed into a lamellar phase, leading to uni- and multilamellar liposomes. At a mass ratio of 3/7 and a similar pH interval, cubosomes of the *Pn3m* type were identified at pH 2.0, 3.0 and 3.5, while primitive *Im3m* type cubosomes formed at pH 4.0, and a lamellar phase at pH 4.5 and over. Thus, the study evidenced a significant pH-responsive effect on the organization of the lipid’s bilayers owing to the integration of 2OHOA. The structural changes upon increasing pH from 2.0 to 7.4 were attributed to the charge repulsion between the gradual deprotonation of carboxylic groups of 2-hydroxyoleic acid embedded at the lipid-water interfaces. The spontaneous curvature of the bilayers was modified due to the increase of the polar headgroup areas (Figure 3c). The obtained nanostructures at different pH values were identified by means of cryo-TEM imaging as well (Figure 3e).

pH-sensitive cubic lipid phases were studied by Nazaruk et al. [64] as soft matter drug delivery matrices. In this work, surfactants were not used for cubosome preparation. pH-dependent drug release from bulk liquid crystalline structures was achieved in the presence of a synthetic lipid bearing two carboxylate groups on the polar head (“Lipid 1”) [65]. The charge of the hydrophobic/hydrophilic interfaces in the liquid crystalline phases was dependent on the protonation/deprotonation state of the new “Lipid 1” under specific pH conditions, which thus modulated the release of the incorporated drug doxorubicin. Thereby, the drug release was faster at pH 7.5 than at pH 5.8, presumably due to the electrostatic attraction between the negatively charged headgroup of “Lipid 1” and the positively charged doxorubicin at the lower pH value. The opposite effect was found in the absence of “Lipid 1”, as summarized in the following section.

Oka et al. [66] used dioleoylphosphatidylserine (DOPS) in a mixture with monoolein at a 2/8 molar ratio to obtain a lamellar phase at neutral pH, which undergone a transition to a primitive cubic *Im3m* phase upon a decrease of pH below 2.75. The authors unveiled by time-resolved SAXS an initial fast transition from a lamellar phase to an inverted hexagonal H_II_ phase followed by a subsequent slow transition to a cubic phase. The effect of electrostatic interactions on the free energy of the transition state was discussed for the charged lipid membranes at different pH values [67,68]. 

Recently, the release of the positively charged drug doxorubicin from lipid cubic phases was controlled by the introduction of appropriately charged lipids as dopants of the host MO lipid bilayers. In this sense, Nazaruk et al. [69] included negatively or positively charged derivatives of monoolein (oleoyl-*rac*-glycerol) into matrix liquid crystalline structures. This allowed modulating the electrostatic interactions with the encapsulated doxorubicin, which is positively charged under acidic conditions (see the structure in Figure 2). At pH 5.5, doxorubicin release was faster in the presence of *N*-(2-aminoethyl) oleamide (CL), but slower in the presence of *N*-oleoyl-glycine (AL) as compared to the pure monoolein-based *Pn3m* cubic phase. These results were ascribed to electrostatic interactions of the guest lipids CL and AL with doxorubicin. Indeed, the positively charged ammonium groups of CLs can repel doxorubicin at pH 5.5, while the negatively charged carboxylate groups of AL can promote the attractive electrostatic interactions with the drug. At variance, the drug release was slower at pH 7.5 when the doxorubicin molecules are mainly in a neutral form. Evidently, a highly hydrophobic structure must be inserted in the apolar regions of the lipid’s bilayers at pH 7.5.

In terms of packings into a cubic structure, the incorporation of the protonated and positively charged lipid CL (at pH 5.5) increased the negative curvature of the amphiphilic bilayers, which resulted in a cubic phase formation with a smaller unit cell as compared to that of the pure monoolein system [69]. In contrast, a slight increase in the cubic lattice parameter was evidenced with the negatively charged AL, which was ascribed to the electrostatic self-repulsion between the lipid head groups. Notably, the lipid cubic phase *Pn3m* symmetry was not altered upon the addition of the charged lipids at the two pH values. This indicated that the pH-dependent release is triggered by electrostatic forces.

**Figure 4:**
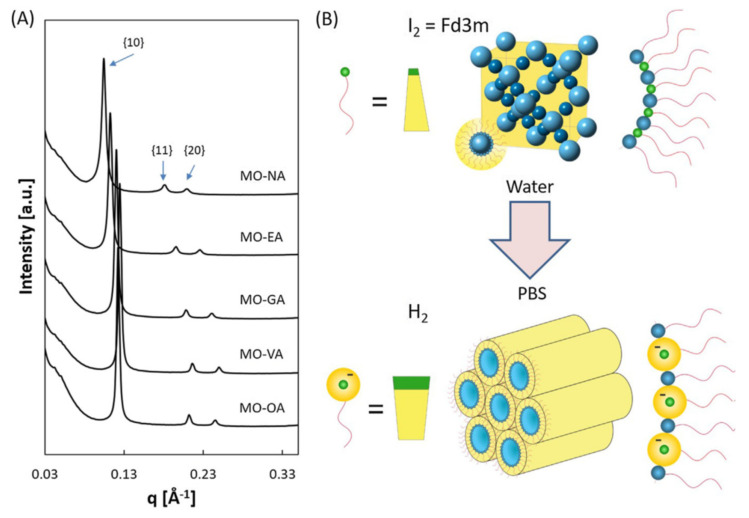
(**A**) SAXS patterns of monoolein (MO)-fatty acid nanoparticles exhibiting a hexagonal H_II_ symmetry in buffer medium (PBS, pH 7.4) at 30 °C. (**B**) Schematic presentation of the effect of pH on the ionization state of the unsaturated fatty acid and the interfacial curvature leading to a transformation of the micellar cubic *Fd3m* phase into an inverted hexagonal H_II_ phase when water is replaced by PBS (reproduced from [70], with permission from Elsevier, 2020).

Recently, Fong et al. [70] reported a comprehensive SAXS study of temperature- and pH-sensitive cubic phases of monoolein individually incorporating oleic acid (OA), vaccenic acid (VA), gondoic acid (GA), erucic acid (EA) or nervonic acid (NA), i.e., five unsaturated fatty acids (containing a single *cis* double bond) with hydrocarbon chain lengths between 18 and 24 carbons. In this study, a micellar *Fd3m* cubic phase was produced in water at pH around 4.9 and a very low ionic strength. In the PBS buffer medium, the elevated pH and higher ionic strengths exerted significant effects on the self-assembled lipid structures. Since the guest fatty acids are characterized by p*K*_a_ around 4.9, the carboxylic acid headgroups deprotonate and acquire a negative charge at pH 7. Hence, the charge repulsion between the headgroups causes a decrease in the interfacial curvature, which results in a phase transition from a cubic *Fd3m* to an inverted hexagonal (H_II_) structure (Figure 4). Noteworthy, the increased ionic strength in PBS has an opposite effect on the interfacial monolayer curvature [32,33]. The authors concluded that the pH change overwhelmed the effect of the increased ionic strength, thus evidencing the pH-sensitivity of the studied systems [70].

**Figure 5:**
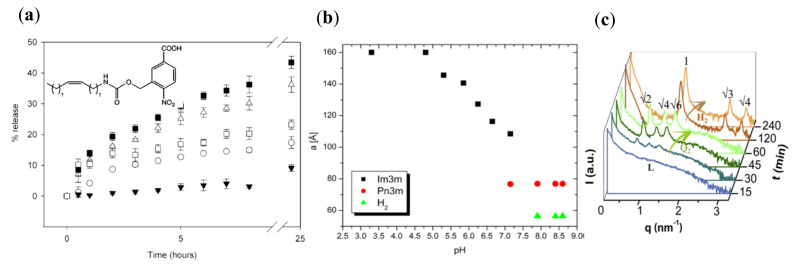
(**a**) Percentages of guest molecule MG (methylene green zinc chloride double salt) release as a function of time from the aqueous compartments of the following five lipid cubic phases: monoolein, pH 7 (white squares); monoolein/Lipid 3 (inserted), pH 7 (black triangles); monoolein/Lipid 3, pH 5 (white circles); monoolein/Lipid 3, pH 3 (white triangles); monoolein/Lipid 3, pH 2.5 (black squares; reproduced from [71], with permission from Wiley-VCH Verlag GmbH & Co., 2020). (**b**) Lattice parameters as a function of pH for monoolein-nicergoline dispersions (reproduced from [72], with permission from the American Chemical Society, 2020). (**c**) Time-resolved SAXS profiles showing phase transitions of lipid dispersions induced by a guest molecule (DBC), which hydrolyzes from neutral to alkaline media (reproduced from [75], with permission from Elsevier, 2020).

Another cubic phase, functionalized by a specially designed lipid, was investigated by Rahanyan-Kägi et al. [71]. The monoolein cubic phase was doped with 1 wt % of a novel synthetic “Lipid 3” containing a *p*-nitro benzoic acid head group (Figure 5a, inset) with p*K*_a_ around 3.6. The incorporation of “Lipid 3” rendered the cubic *Pn3m* liquid crystalline phase pH-sensitive. The release of encapsulated hydrophilic cationic compound MG (methylene green zinc chloride double salt) was monitored as a function of various pH conditions. The deprotonation of the carboxylate head group of the guest lipid at pH 7 resulted in binding with the positively charged MG, in contrast to the pure monoolein lipid phase, in which the head groups are neutral. Upon the decrease of the pH to 5, 3 and 2.5, the carboxylate head group of “Lipid 3” was progressively protonated. This led to losing the charged character, and thus to decreased binding between MG and “Lipid 3”. As a result, an increased MG release from the cubic phase over 40% was attained after 24 h, whereas the release was around 20% from the pure monoolein system (Figure 5a). The study showed the potential of the liquid crystalline structures containing the synthetic “Lipid 3” as a platform for the development of drug delivery nanomaterials with pH-controlled release properties.

## 3. Drugs as pH-Responsive Inducers

Partitioning of guest molecules between the lipid’s bilayers and the aqueous domains in amphiphilic cubic phase networks is crucial for the release rate of the incorporated drugs and bioactives, which can be furthermore pH-dependent.

The anti-cancer drug doxorubicin (DOX) behaves as an electro-active molecule due to the presence of quinone and hydroquinone groups (Figure 2). Nazaruk et al. [64] monitored the electrochemical behavior of doxorubicin incorporated in monoolein and phytantriol cubic phases under different pH conditions. Doxorubicin charge is pH-dependent considering the p*K*_a_ value of 8.2. The study suggested that the drug resides mainly in the cubic lattice aqueous channels at acidic pH 5.8 provided that DOX is 99.6% protonated. Therefrom, the drug release from the cubic lipid structure at acidic pH was faster in comparison to the alkaline conditions. At pH 7.5, it was reported that 16.6% of doxorubicin is uncharged and that this fraction increases to 87% at pH 9.0. The uncharged molecules are thus expected to reside mainly in the hydrophobic domains of the lipid membranes. Hence, the DOX release rate was considerably reduced under alkaline conditions due to the entrapment of the uncharged drug in the lipid cubic phase. Later, Nazaruk et al. [82] further confirmed by molecular dynamic simulations that protonated doxorubicin is mainly located in the aqueous channels of the cubic phase, whereas the unprotonated form interacts preferentially with the hydrophobic domains of the bilayers. The authors emphasized that such a pH dependence of the DOX release rate from the cubic phase may be exploited for controlled drug release into tumor cells, where the pH is lower than that of healthy cells (see also Figure 3a).

Salentining et al. [72] studied cubosomes produced by ultrasonication of monoolein and nicergoline, a poorly water-soluble vasoactive drug applied for treatments of dementia, vascular and balance disorders [83]. The drug was successfully incorporated in the lipid bilayers at proportions from 10% to 30% *w*/*w*. The addition of nicergoline modified the phase behavior of the liquid crystalline structures and rendered a strongly pH-responsive system. The three amine groups in the molecule, acting as hydrogen acceptors, increasingly protonated at pH values below and near the p*K*_a_, leading to positive charges and a more amphiphilic character of the drug. At pH > 8, a coexistence of *Pn3m* cubosomes and hexosomes was unveiled by SAXS for 10% *w*/*w* nicergoline content (Table 1). At pH 7.2, a coexistence of *Pn3m* and *Im3m* bicontinuous cubic phase structures was identified. The decrease of pH from 7.0 to 3.3 led to the vanishing of the *Pn3m* cubic phase, and a gradual increase of the lattice constant of the *Im3m* phase from 105 to 160 Å (Figure 5b). The increase of the drug concentration provoked further structural transitions under different pH conditions, e.g., from emulsified microemulsion at higher pH values to vesicles formation under acidic conditions. The authors discussed that protonation of the nicergoline molecules in the bilayers leads to an increase in the effective area of the hydrophilic moieties, thereby promoting the observed phase transitions [72].

Zabara et al. [73] produced monolinolein cubic phases embedding the outer membrane protein F (OmpF), which is a major component of the outer membrane of the Gram-negative bacteria *Escherichia coli*. A perforated *Pn3m* cubic phase was generated with the inclusion of OmpF within the lipid bilayers. It was demonstrated that the on-off opening of the perforating pores is mediated by the response of the membrane protein to pH variations. At a physiological pH of 7.4, it was shown that the interconnecting of the aqueous channels of the *Pn3m* cubic phase via the OmpF pores (present at a very low concentration of 0.1 wt %) significantly influences the release rate of encapsulated glucose from the bulk cubic phase. It increased to 22% in the presence of the membrane protein. At pH 4.8, the OmpF protein-doped and the protein-free cubic phases showed identical release profiles, indicating that the protein blocked the exchange between the aqueous channels under acidic conditions. This effect resulted in the same release kinetics profiles of glucose. Since the OmpF pore contains a constriction site with two amino acid half rings (one positively and one negatively charged) resulting in an electrostatic field, this efficiently modulated the solute fluxes and the nanopore properties in response to changes in the environmental pH. Thereby, it was demonstrated that the electrostatic field acts as a natural gate under acidic conditions and blocks the transport across the protein pore. Alternatively, the protein allows free exchange with pore opening at higher pH. The pH-mediated response of the double amino acid half-ring architecture of the membrane protein provided a mechanism, in which the pores of the perforated mesophase can be opened or closed via a pH trigger. The latter allows modulation of the transport of encapsulated molecules as well as the release properties by moderate changes in pH.

Yaghmur et al. [74] studied the pH-responsive effect of bupivacaine, a local anesthetic drug, on the liquid crystalline structures of hydrated monoolein and monolinolein doped with medium-chain triglycerides (caprylic and capric acids). Bupivacaine is a weak base with a p*K*_a_ value of about 8.1 at 37 °C [84]. The study showed that the release rate of the drug from the liquid crystalline phase was significantly faster at pH 6.0 as compared to that observed at pH 7.4. It was discussed that the impact of pH on the release rates is likely related to the higher affinity of the drug to the lipidic phase at pH 7.4, which triggered the formation of a highly curved H_II_ phase. Indeed, the increased incorporation of bupivacaine into the lipidic matrix induced a structural transition from a *Pn3m* cubic phase to an H_II_ phase at pH 7.4. At variance, a complete transition was prevented at pH 6.0 for the investigated drug loading. It was reported that 99.2% of bupivacaine is ionized at pH 6.0, whereas the fraction of its uncharged form increases to 16% at pH 7.4. The latter led to a relatively higher amount of drug entrapped in the hydrophobic region of the lipid membranes. On the other hand, the increased level of bupivacaine ionization with a decreasing pH from 7.4 to 6.0 enhanced the preferential localization of the drug molecules in the hydrophilic nanochannels of the self-assembled systems, thereby favoring faster drug release.

## 4. Ionic Surfactants

The inclusion of ionic surfactants into cubic-phase-forming binary lipid/water systems may influence their phase behavior and their structural properties, such as bilayer thickness, water channel diameter and interfacial curvature [85], resulting in amphiphile self-assembly which may feature specific pH-responsive characteristics.

Ribeiro et al. [75] investigated the effect of decyl betainate chloride (DBC) inclusion in phytantriol-based dispersions and established a gradual lamellar-to-inverted cubic-to hexagonal H_II_ phase transition under physiological pH conditions. Cubosomes of *Pn3m* symmetry with incorporated 3.6% (*w*/*w*) DBC in the lipid bilayers were obtained by ultrasonication. However, a higher concentration (10% *w*/*w*) of DBC yielded niosomes. Peculiarities of the colloidal dispersions were observed under different pH conditions. For instance, the dispersions kept the initial structural organization in acidic media. Notably, DBC cleavage occurred via hydrolysis in neutral and alkaline media producing 1-decanol (which remained in the lipid bilayer) and betaine hydrochloride (freely solubilized in the aqueous solution). Consequently, the formation of 1-decanol induced lipid bilayer packing with greater curvature. The time dependence of the phase transitions was examined by SAXS at pH 7.4 (Figure 5c). It was evidenced that niosomes (L phase) transform into particles of *Im3m* cubic symmetry followed by a transition to an H_II_ phase structure. Thus, the association of DBC to phytantriol turned out to be promising for the production of pH-responsive drug carriers with applications in oral drug delivery. Indeed, the phase transitions induced in the alkaline medium of the gut may be expected to provide pH-triggered drug release.

Hubcik et al. [76] added different concentrations of *N*,*N*-dimethyldodecylamine-*N*-oxide (C_12_NO), a cationic surfactant, to a DOPE host lipid phase and DNA aiming at the fabrication of a pH-sensitive system for gene delivery. The authors exploited the fact that the polar fragment of C_12_NO (which is charged under acidic conditions) can interact with the polar head of DOPE yielding a lateral expansion of the lipid bilayer interface. On the other hand, the mismatch between the C_12_NO alkyl chain length substituent (inserted in the bilayer) and the length of hydrocarbon acyl chains of DOPE (18 carbons) can create defects in the hydrophobic membrane core, hence leading to polymorphism of the lipid bilayers. For a particular composition at pH ~7, the amphiphilic mixture formed a *Pn3m* cubic phase when the complexes were heated to 80 °C and then cooled down to 20 °C. Under acidic conditions, a condensed inverted hexagonal H_II_ phase was reported, besides the formation of a lamellar phase. Both the lipid composition and pH affected the DNA binding in the complexes. The strongly pH-dependent polymorphic behavior provided a large variety of liquid crystalline phases. It was concluded that the surfactant–lipid mixtures enable the efficient uptake and release of the anionic DNA macromolecules and thus are promising for the development of C_12_NO/lipid-based nanomaterials for the design of delivery vectors of genetic material [76].

Astolfi et al. [85] recently evaluated the effect of the cationic surfactant didodecyldimethyl-ammonium bromide on phytantriol cubic phases and particle dispersions. Although the systems were not tested under different pH conditions, it is worth mentioning that the increased surfactant concentration in the mixtures can induce a transition from a cubic *Pn3m* to a cubic *Im3m* and lamellar phases. Moreover, the encapsulated anionic anti-cancer drug 5-fluorouracil stabilized the *Pn3m* cubic phase at all investigated cationic surfactant concentrations. However, the neutral form of the drug did not cause such an effect. In fact, the presence of the cationic surfactant in the cubic mesophase structure increases the surface charge density. Thereby, the enhancement of the electrostatic repulsions between the charged lipid headgroups provokes a decrease of the negative interfacial curvature, and therefore the occurrence of a phase transition. It remains to be unveiled how these characteristics will be influenced by different pHs and by the presence of charged guest molecules.

## 5. Polyelectrolytes in Cubosomes

The use of polymers, polyamino acids and proteins containing ionizable amine and carboxylic groups, and undergoing protonation–deprotonation in specific pH ranges, has recently emerged as a strategy for the development of cubosome nanocarriers and cubic phases with pH-responsive characteristics. 

**Figure 6:**
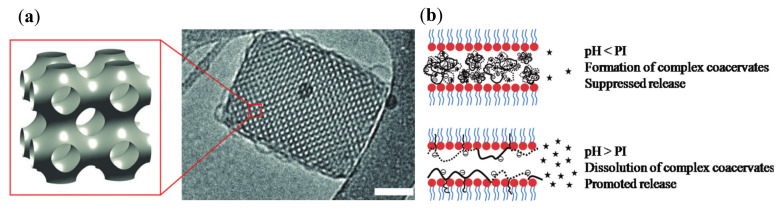
(**a**) Three-dimensional (3D) presentation of a primitive *Im3m* cubic lipid lattice and a cryo-TEM image of PP50 polymer-modified monoolein cubosome (reproduced from [77], with permission from the Royal Society of Chemistry, 2020). (**b**) Scheme of the drug release (stars) from the water channels of a monoolein cubic phase modified by polyelectrolytes (reproduced from [80], with permission from the American Chemical Society, 2020).

Kluzek et al. [77] produced pH-sensitive cubosomes by the assembly of monoolein with poly(*L*-lysine-*iso*-phthalimide) grafted with *L*-phenylalanine at a grafting degree of 50% (PP50). The pH-sensitive polymer was incorporated at 10 wt % in the cubosomes stabilized by the nonionic Pluronic F127 surfactant. The hydrophobic/hydrophilic balance of the polyelectrolyte and its conformational flexibility caused a partial distortion of the *Im3m* cubosome structure at pH 5.5, while the integrity of the structure was preserved at pH 7.5 (Figure 6a). Partially or totally disordered particles were found to coexist with ordered cubic and lamellar phase particles only under acidic conditions. It was emphasized that the partial disappearance of the cubic phase under acidic conditions may render the pH-sensitive cubosomes a promising formulation for drug delivery applications.

Chountoulesi et al. [78] combined poly(2-dimethylamino ethyl) methacrylate (PDMAEMA), a cationic polyelectrolyte, in a conjunction with the hydrophobic poly(lauryl methacrylate) (PLMA), to produce a pH-responsive cubosome in association with monoolein or phytantriol. The cationic polyelectrolyte bears an ionizable tertiary amino group, which is protonated under acidic conditions. The polymer exhibits a conversion from hydrophilic to hydrophobic character under pH increase resulting from its deprotonation and the involved polymer–polymer interactions [86]. A copolymer PDMAEMA-*b*-PLMA was synthesized and incorporated in lipid cubosomes prepared by the top-down method. Taking advantage of the proton buffering capacity of PDMAEMA, different pH-dependent cubic and vesicular structures were obtained in a variety of formulations. The charge of the self-assembled systems was pH-responsive, thus indicating the exposure of the polymer chains over the particles’ surface.

Seo and Kim [79] employed a peptide composed of aspartic acid and leucine as an acidic proteinoid and poly-lysine as a basic proteinoid in order to produce a monoolein-based cubic phase permitting pH-dependent release of encapsulated probes. The proteinoids were prepared by the melting-condensation method. Heating of dicarboxylic amino acids led to the thermal condensation of acidic proteinoids, whereas thermal polymerization of basic amino acids yielded basic proteinoids (e.g., poly-lysine). The proteinoids were incorporated in the hydrated monoolein bulk phase along with FITC–dextran. It was suggested that the proteinoids are embedded in the water channels of the cubic network, despite that a cubic symmetry was not identified by structural methods. A complexation between the proteinoids occurred through electrostatic interactions at pH 7.0, at which the acidic proteinoid and the basic proteinoid were inversely ionized. Under acidic conditions, the acidic proteinoid was mostly in a unionized form. Therefore, the intermolecular electrostatic interactions were relatively weak. Contrarily, the basic proteinoid tends unionize in an alkaline medium, thus weakening the electrostatic interactions. Thereby, the maximum complexation was attained at a neutral pH. The release study was undertaken for 40 h. It revealed a relatively suppressed release of FITC–dextran from the composite cubic phase under a neutral pH condition, while the release increased at both acid and alkaline pHs. The obtained effects were ascribed to the pH-dependent complexation of the proteinoids in the water channels of the lipid cubic phase, which acted as a controlled release carrier in response to changes in the pH of the medium.

Kwon and Kim [80] utilized a hydrophobically modified alginate along with a hydrophobically modified silk fibroin to functionalize the monoolein cubic phase. Although the cubic phase was not characterized by structural methods, its association with both polyelectrolytes provided the pH-dependent release of the incorporated fluorescent probe, FITC–dextran. Since the modified alginate presents stearylamine groups and the modified fibroin presents succinic acid residues, they can actively modulate the release in a pH-dependent manner. Fibroin is positively charged at pH below the isoelectric point (PI). Therefore, the electrostatic interaction with the negatively charged alginate leads to the formation of a complex coacervate, which in practice can block the water channels of the cubic phase, hence hindering the release at low pHs. Upon increasing the pH of the medium above the isoelectric point, fibroin becomes negatively charged and then the coacervates dissolve. This allowed the enhanced release of encapsulated FITC–dextran (see the scheme in Figure 6b). More recently, Park and Kim [87] described similar effects by replacing silk fibroin to a hydrophobically modified gelatin. 

Crisci et al. [81] described the synthesis of a polyacid-lipid by conjugation of poly-(acrylic acid) (PAA) with 1,2-dimyristoyl-*sn*-glycero-3-phosphoethanolamine (DMPE). The fabricated liquid crystalline assemblies behaved as pH- and ionic strength-responsive materials. The conjugate was included at 8–11 mol % with DMPC lipid and *N*,*N*-dimethyl-dodecylamine *N*-oxide as a co-surfactant in the complex mesophases. Under strong acidic conditions, the covalently-grafted acrylic acid chains were assumed to adopt a coiled conformation [88]. Besides, the protonated state of the PAA chains may favor hydrogen bonding with the phosphodiester group of the lipids, thus leading to a strong association with the lipid membrane. It was argued that these characteristics can produce sufficient steric repulsion within the lamellae, thus yielding a swollen lamellar structure as identified by SAXS. Mixtures of swollen and dehydrated (collapsed) lamellar phases were observed at pH 3.8 in the presence of salt. It was attributed to the decrease in the polymer layer thickness under salted conditions, where the electrostatic screening is strong [89]. Under these conditions, the grafted polymer must be more tightly coiled and associated with the lipid headgroup region, providing reduced steric repulsion between the opposing lamellae. Thereby, the water layer thickness decreased, and accordingly, the interlamellar repeat distance in the swollen lamellar phase was reduced. The obtained lamellae were referred to as “collapsed lamellae”. A multilamellar phase coexisting with a collapsed lamellar structure was described near the neutral pH 6.8. The partial phase reorganization was ascribed to the subtle expansion of the coil conformation of the incompletely ionized PAA. However, a primitive cubic phase was produced at pH 9.8 along with the collapsed lamellae. It was pointed out that the deprotonated, and thus completely negatively charged polymer chains, adopt an extended conformation under alkaline and moderate ionic strengths. Such an uncoiled polymer conformation should have driven the formation of a cubic mesophase of network organization. Thereby, the state of ionization of the embedded PAA and its conformation state at different pH values induced significant changes in the host lipid matrix.

## 6. Perspectives of pH-Sensitive Cubosomes

The notable pH-sensitive behavior of cubosomes and bulk liquid crystalline cubic phases represents high technological appeal in the development of specific structure-responsive drug delivery nanomaterials. 

**Figure 7:**
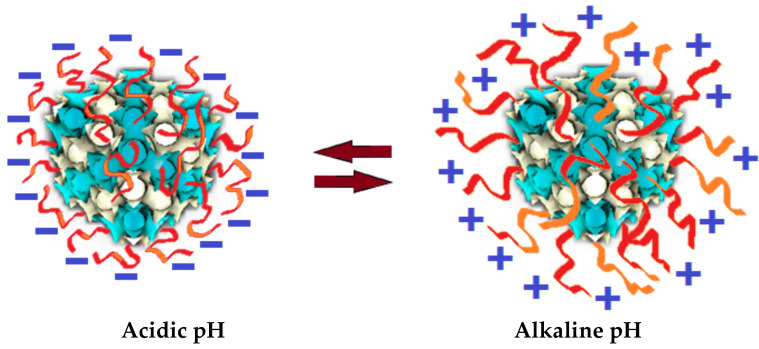
Concept of composite polyelectrolyte/nonlamellar lipid cubosome nanocarriers of pH-sensitive structures and modulable charges enabling electrostatic swelling of the supramolecular architectures and providing new possibilities for drug loading and controlled release.

The recently reviewed literature suggests various research directions, in which new pH-sensitive cubosomes are prone to be produced with the synthesis of specially designed lipids and guest additives. The prospective functionalization strategies aim at improved performance of the cubosome drug delivery carriers under the application of environmental stimuli, where the pH variations play an important role (Figure 7). The custom chemical synthesis of charged lipids, strategically designed [65], represents a plethora of means for the purposeful production of cubosomes for many specific drug delivery applications besides the gene delivery studies [90,91,92]. Whereas cubosomes were previously sterically stabilized by PEGylated lipids and non-ionic copolymer shells [93,94], more recent research directions tend to combine the advantages of both lipid and polymer derivatives [95,96,97,98,99,100,101,102]. 

Furthermore, the production of composite cubosomes and cubic phases by associating lipids with macromolecules of pH-dependent structure, including large proteins, polypeptides, polymers, polysaccharides and peptide conjugates [21,97,103,104], can provide a wider range of novel delivery systems [105,106,107,108,109,110,111,112]. 

Dual functional composite cubosomes may further be envisaged in employing chemically modified macromolecules able to render (i) specific pH-responsive characteristics; (ii) additional biological benefits such as nutraceutical or anti-oxidant effects; (iii) auxiliary improvement of drug penetration into cells; (iv) activation or promotion of drug release at the targeted site; or (v) boosters of pharmacological effect of drugs, vaccines and transfection agents of plasmid-DNA or siRNA in cell lines. The phase behavior of such pH-stimuli responsive systems can be further exploited for the elaboration of mucoadhesive controlled release formulations needed for mucoadhesive prolonged-release applications [113,114,115,116,117]. 

In this context, well-known macromolecules of biocompatible and biodegradable nature and of low immunogenicity are of high scrutiny [45,46,95,96]. This is the case of synthetic polyethylene glycol, polylactic acid and polycaprolactone, and natural source chitin, chitosan and alginate [54,93,94,97,99,102]. For instance, chitosan, which is a polysaccharide containing protonable amino groups was largely explored in an association with lipids for the production of composite bioactive delivery systems [46,47,52]. Furthermore, cubic phases were functionalized [45] and the transition between the mesophase structures of cubic symmetries was dependent on the chitosan concentration and the impact of cationic charge effects [46]. Hence, the electrostatic energy may be further explored in the production and application of pH-sensitive cubosomes [25,66,69]. 

In terms of chemically modified macromolecules, aiming at improved biological performance, chitosan derivatives modified with anti-oxidant flavonoids showed improved anti-oxidant effects over lipid membranes [101]. More recently, the modification of chitosan with arginine (i.e., chitosan-*N*-arginine) and the complexation of the biopolymer in liposomal carriers with pDNA, forming lipo-polyplexes, demonstrated enhanced transfection efficiency [102]. The improved outcome was ascribed to the cell-penetrating characteristics of arginine, which is a positively charged amino acid under all physiological conditions.

On the other hand, the association of chitosan with alginate as well as of chitosan-*N*-arginine with alginate was employed in the production of pH-responsive micro-nano-particles for oral drug delivery [46,52]. Indeed, the association of the two biopolymers presented dual advantages. First, the positive charges of the protonated chitosan and the negative charges of the carboxylic groups of alginates provided profitable pH-responsiveness of the obtained complexes at pHs of the gastrointestinal tract. Second, their muco-adhesiveness with the intestinal mucosa, allowing interaction and prolonged retention of the particles in fish intestine, was emphasized as a highly desired feature for improvement of localized, prolonged and sustained oral drug delivery.

These studies have expanded the opportunities for the development of pH-sensitive cubosomes, where the lyotropic nonlamellar lipids and the encapsulated drugs can be protected by pH-sensitive polymer shells. Concerning oral drug delivery, composite pH-responsive cubosomes may be developed in order to increase the chemical and the structural stability of the drug delivery dispersions in the harsh acidic conditions of the stomach, while promoting the mucoadhesive characteristics at the targeted site of drug release (which is usually the intestinal tract where the absorption of drugs takes place). The improved performance of such drug delivery dispersions can profit from adequate charge-charge interactions, as for instance, between the positively charged polycations on the cubosomes surface and the negative carboxyl charges of sialic acid groups of mucins (see the scheme of the large transmembrane protein, which covers the mucosal cell membranes in Figure 2). In this case, the electrostatic interaction may provide the prolonged retention of the cubosome nanostructures, and thereby allow the in situ drug release, which can be further modulated by the phase transitions of cubic symmetry in a pH-responsive manner. 

In conclusion, as reviewed in this article, a variety of strategies for engineering cubosomes can be envisioned in order to render these liquid crystalline structures advanced drug delivery nanomaterials taking advantage of the pH changes of the biological media at targeted application sites. 

## Figures and Tables

**Table 1:** Summary of reported compositions and main characteristics of self-assembled pH-responsive liquid crystalline mesophases and nanoparticles derived thereof.

Lipids	Additives	Preparation Methodology	Studied pH Values	Liquid Crystalline Phases	Perspective for Application	Refs.
Monoolein Oleic acid	*Brucea javanica* oil Pluronic F127 PBS Doxorubicin	Melting 60 °C Stirring High-pressure homogenization	7.4 6.8 5.3	H_II_ *Pn3m*, *Im3m* microemulsion	Dual-drug (BJO, DOX) delivery/cancer inhibition (in vitro tested)	[59]
Monoolein Oleic acid	Pluronic F127 PBS	Heating 80 °C Homogenization High pressure	6.0 7.0	H_II_ *Im3m*	Drug delivery (perspective)	[60]
Monolinolein Linoleic acid	Phloroglucinol	Hydration Heating Vortex mixing	2.0 7.0	H_II_ *Im3m*	Oral drug delivery (perspective)	[61]
Monolinolein Pyridinylmethyl linoleate	Doxorubicin	Hydration Heating Vortex mixing	5.5 7.4	*Pn3m* H_II_	Tumor-targeted delivery (in vitro tested)	[62]
Monoolein 2-hydroxyoleic Acid	Pluronic F127 PBS	Ultrasonication	2.0; 3.0 3.5; 4.0; 4.5 5.0; 6.0; 7.4	*Pn3m*, H_II_ *Pn3m*, *Im3m* Lamellar	Tumor-targeted delivery (perspective)	[63]
Monoolein Phytantriol “Lipid 1”	Doxorubicin	Melting Hydration Centrifugation	5.8 7.5 9.0	*Pn3m* *Pn3m* *Pn3m*	Drug delivery (perspective)	[64]
Monoolein DOPS	-	Hydration Vortex mixing Centrifugation	6.7 2.75 2.55	L H_II_ *Im3m*	Drug delivery (perspective)	[66]
Monoolein *N*-Oleoyl-glycine *N*-(2-aminoethyl)-oleamide	Doxorubicin	Melting Hydration Centrifuge mixing	5.5 7.5	*Pn3m* *Pn3m*	Drug delivery (perspective)	[69]
Monoolein Oleic acid Vaccenic acid Gondoic acid Erucic acid Nervonic acid	Pluronic F127 PBS	Hydration Ultrasonication	4.9 7.0	*Fd3m* H_II_	Drug delivery (perspective)	[70]
Monoolein “Lipid 3”	Methylene green zinc chloride double salt	Hydration Centrifugation	2.5 3.0 5.0 7.0	*Pn3m* *Pn3m* *Pn3m* *Pn3m*	Drug delivery (perspective)	[71]
Monoolein	Nicergoline Pluronic F108	Ultrasonication	3.3; 5.6; 5.9; 6.7 7.2 8.4	*Im3m* *Im3m* *Pn3m*, *Im3m* *Pn3m*, H_II_	Drug delivery (perspective)	[72]
Monolinolein	“Outer membrane protein F”	Heating 45 °C Vortex mixing	4.8 7.4	*Pn3m* *Pn3m*	Drug delivery (perspective)	[73]
Monoolein Monolinolein	Bupivacaine Caprylic acid Capric acid	Heating 50 °C Hydration Heating 60 °C Vortex mixing Incubation at 37 °C (1–2 weeks)	6.0 7.4	*Pn3m* H_II_	Drug delivery (perspective)	[74]
Phytantriol	Pluronic F127 Decyl betainate chloride	Ultrasonication	3.9; 5.5 7.4; 8.5	*Pn3m*, L *Im3m*, H_II_	Oral drug delivery (perspective)	[75]
DOPE	DNA *N*,*N*-dimethyldodecyl- amine-*N*-oxide	Hydration Vortex mixing Freeze–thaw	7.2 4.8	H_II_, L, *Pn3m* H_II_, L	Genetic and drug delivery (perspective)	[76]
Monoolein	PP50 ^1^ Pluronic F127	Hydration Sonication Stabilization with surfactant	7.5 5.5	*Im3m* *Im3m*, swollen	Drug delivery (perspective)	[77]
Monoolein Phytantriol	Poloxamer P407 PDMAEMA-*b*- PLMA	Hydration Ultrasonication	4.2 6.0 7.4	*Im3m*, L *Im3m*, L *Im3m*, L	Drug delivery (perspective)	[78]
Monoolein	Aspartic acid-leucine peptide Poly-lysine FITC–dextran	Melting 65 °C Hydration	3.0; 5.0; 7.0; 8.5	Not identified	Drug delivery (perspective)	[79]
Monoolein	Modified alginate Modified silk fibroin FITC–dextran	Melting 60 °C Hydration	3.0; 4.0; 4.5; 5.0; 7.0; 9.0	Not identified	Drug delivery (perspective)	[80]
DMPC DMPE	*N*,*N*-dimethyl- dodecylamine- *N*-oxide Poly(acrylic acid)	Hydration Repeated heating 60 °C, vortex mixing, ice bath cooling	<2 3.8 6.8 9.8	L (swollen) L (swollen + collap) L (collap + multiL) *Im3m*, L (collap)	Therapeutic agent (perspective)	[81]

^1^ PP50: poly(*L*-lysine-*iso*-phthalimide) chain grafted with *L*-phenylalanine.
